# Decoding Human Biology and Disease Using Single-cell Omics Technologies

**DOI:** 10.1016/j.gpb.2023.06.003

**Published:** 2023-09-20

**Authors:** Qiang Shi, Xueyan Chen, Zemin Zhang

**Affiliations:** 1 Biomedical Pioneering Innovation Center, School of Life Sciences, Peking University, Beijing 100871, China; 2 Peking-Tsinghua Center for Life Sciences, Academy for Advanced Interdisciplinary Studies, Peking University, Beijing 100871, China; 3 Changping Laboratory, Beijing 102206, China

**Keywords:** Single-cell omics, Computational method, Cellular heterogeneity, Disease, Cancer research

## Abstract

Over the past decade, advances in **single-cell omics** (SCO) technologies have enabled the investigation of **cellular heterogeneity** at an unprecedented resolution and scale, opening a new avenue for understanding human biology and **disease**. In this review, we summarize the developments of sequencing-based SCO technologies and **computational methods**, and focus on considerable insights acquired from SCO sequencing studies to understand normal and diseased properties, with a particular emphasis on **cancer research**. We also discuss the technological improvements of SCO and its possible contribution to fundamental research of the human, as well as its great potential in clinical diagnoses and personalized therapies of human disease.

## Introduction

The cell acts as the fundamental unit of life. A single zygote gives rise to the entire human body, in which approximately 37 trillion cells of diverse types are highly orchestrated into a variety of tissues, organs, and systems. Traditionally, distinct cell types have been defined in terms of cellular morphology, location, or expression levels of a small number of proteins, which grossly neglects differences in additional molecular layers across cells within a population. The large heterogeneity of cells underlies functional diversity in human biology [1]. Notably, the characteristics of a cell are pertinent not only to its own state, size, or ancestor, but also to its unique niche around and how the cell interacts with adjacent or distant cells [2], [3]. For example, although found in most organs to synthesize the extracellular matrix of connective tissue by producing collagen, fibroblasts perform specialized functions depending on the specific contexts across a broad range of tissues and disease conditions [4]. However, conventional bulk sample sequencing technologies mask the diversity of cells, as exemplified by RNA sequencing, which derives average measurements of gene expression for all cells within an experimental sample [5]. Therefore, understanding human biology and disease at single-cell resolution is imperative.

Since the first single-cell RNA sequencing (scRNA-seq) method arising in 2009 [6], numerous single-cell omics (SCO) sequencing technologies have been developed to characterize cellular properties at different molecular layers, including the genome, epigenome, transcriptome, and proteome [6]. Single-cell multimodal omics sequencing represents the state-of-the-art technology, which can simultaneously depict multiple characteristics of one cell [7]. The revolution in SCO sequencing technology has dramatically expanded our toolbox for investigating biomedical systems in which cells develop along their fates, transition between different states, vary across individuals, and fail in disease [8], [9]. Importantly, SCO sequencing has given rise to high-throughput measurements of linkages between intrinsic genotypes and extrinsic phenotypes at the cellular, tissue, organ, and individual levels [10]. These advances have led to many significant insights in the fields of cancer, development, immunity, regenerative medicine, and plant research. Because of its rapid development and enormous potential, SCO sequencing has twice been selected as the Method of the Year by the journal *Nature Methods* [11], [12].

In this review, we will summarize the developments of SCO sequencing technologies and computational tools, and highlight the representative knowledge brought by SCO sequencing, especially in cancer research. Finally, we will provide concrete prospects for SCO technologies in fundamental research and clinical applications over the next few years.

## Development of SCO sequencing technologies

**Figure 1:**
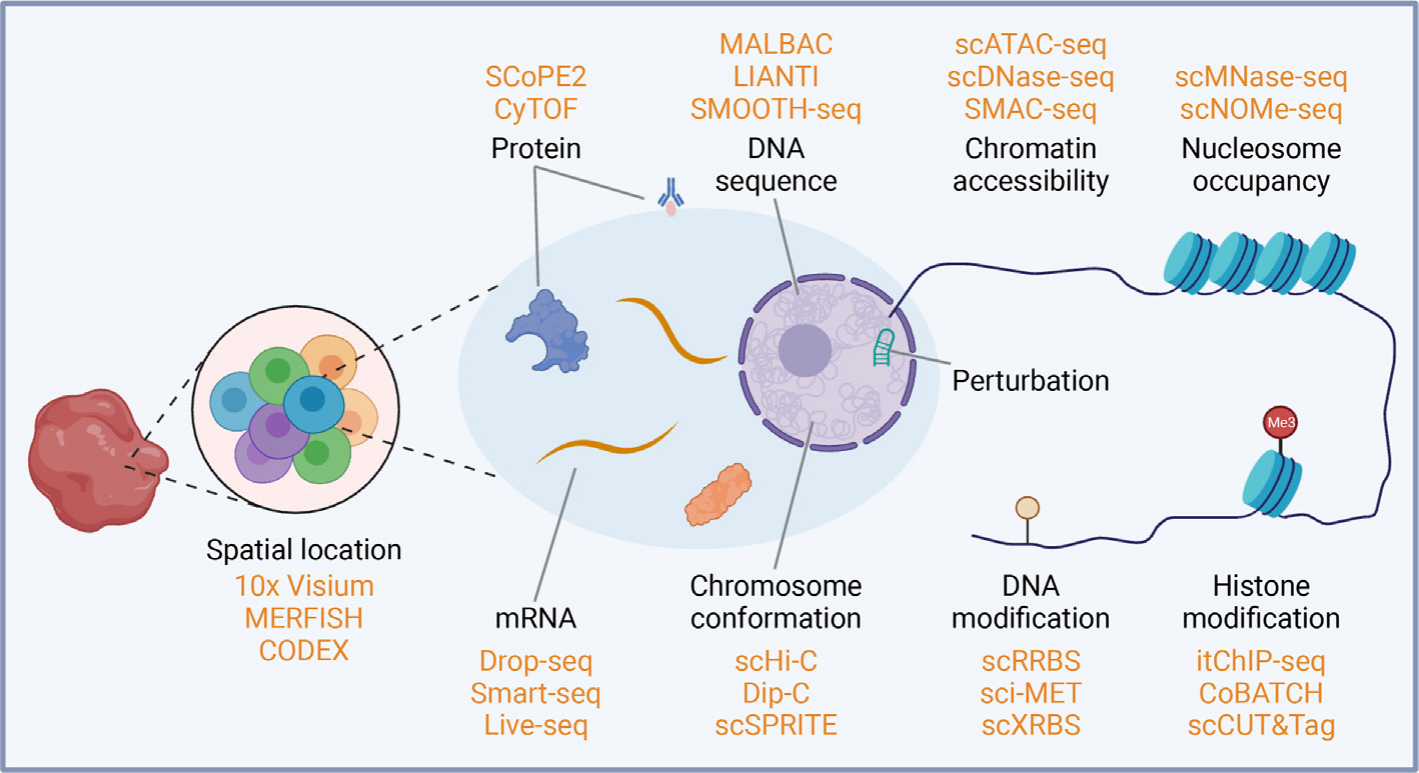
**Cellular heterogeneity at different molecular layers** The cell-to-cell heterogeneity is reflected at several distinct molecular layers. Representative methods for profiling each of the individual molecular levels are noted. Single-cell multimodal omics sequencing technologies have been developed to simultaneously profile multiple layers in the same cell. SCoPE, single-cell proteomics; CyTOF, cytometry by time of flight; MALBAC, multiple annealing and looping-based amplification cycles; LIANTI, linear amplification via transposon insertion; SMOOTH-seq, single-molecule real-time sequencing of long fragments amplified through transposon insertion; scATAC-seq, single-cell assay for transposase-accessible chromatin using sequencing; scDNase-seq, single-cell DNase sequencing; SMAC-seq, single-molecule long-read accessible chromatin mapping sequencing; scMNase-seq, single-cell micrococcal nuclease sequencing; scNOMe-seq, single-cell nucleosome occupancy and methylome sequencing; MERFISH, multiplexed error robust fluorescence *in situ* hybridization; CODEX, co-detection by indexing; scHi-C, single-cell high-throughput chromosome conformation capture; mRNA, messenger RNA; Smart-seq, switching mechanism at 5′ end of the RNA transcript; Dip-C, diploid chromatin conformation capture; scSPRITE, single-cell split-pool recognition of interactions by tag extension; scRRBS, single-cell reduced-representation bisulfite sequencing; sci-MET, single-cell combinatorial indexing for methylation analysis; scXRBS, single-cell extended-representation bisulfite sequencing; itChIP-seq, simultaneous indexing and tagmentation-based chromatin immunoprecipitation with massively parallel DNA sequencing; CoBATCH, combinatorial barcoding and targeted chromatin release; scCUT&Tag, single-cell cleavage under targets and tagmentation.

A primary purpose of SCO sequencing technologies is to disentangle the tremendous cell-to-cell heterogeneity driven by intrinsic programs and extrinsic factors. Essentially, all SCO sequencing technologies aim to decode underlying information surrounding the DNA, RNA, and protein that are the core molecules in the genetic central dogma of molecular biology [13] (Figure 1). In this section, we briefly review the rationales of SCO sequencing technologies at different molecular layers, as their technical details have been recently and elaborately reviewed [14].

### Genetic variants detected by the single-cell genome sequencing

First of all, genomic DNA sequences represent the genetic information of an organism. Diploid cells have only two copies of genomic DNA, posing the main challenge for single-cell whole-genome sequencing. In the last decade, several methods including MALBAC [15], eMDA [16], LIANTI [17], SISSOR [18], and META-CS [19], have been established to improve the uniformity, efficiency, accuracy, and coverage of the whole genome amplification at single-cell resolution. Although most cells contain the same two copies of genomic DNA, plentiful genetic variants of different types, such as single nucleotide variants (SNVs), small indels, copy number variations (CNVs), and structural variations (SVs) caused by stochastic mutations, emerge in low frequency yet gradually accumulate during the development, aging, as well as disease progression across multiple tissue types of the human body [20], [21], [22]. Therefore, these genetic variants detected by single-cell DNA sequencing can be intrinsic markers tracing the historical trajectory of a cell in the human body. In addition, as another part of the cellular genome, mitochondrial DNA (mtDNA) has hundreds of copies in an individual cell and 10–100-fold higher mutation rates than nuclear DNA. Single-cell genomic assays can be used to detect somatic mtDNA mutations and to track cellular clones via routine sequencing [23]. Recently, the SMOOTH-seq, a novel single-cell genome sequencing method based on the single-molecule real-time DNA sequencing platform, has been reported [24]. Compared with methods based on the short-read sequencing platforms, SMOOTH-seq performs better for the detection of SVs and extrachromosomal circular DNA in individual cells, but shows lower accuracy for CNVs and SNVs.

### Revealing the complexity of epigenetic regulations at single-cell resolution

The epigenetic regulation including chromatin states, chromosomal conformations, and DNA or histone modifications, is characterized by heritable variations independent of DNA sequences, representing a crucial molecular mechanism associating the genetic information with its functional output [25]. In eukaryotes, nuclear chromatin is composed of basic repeating structural and functional subunits called nucleosomes, which consist of approximately 146 base pairs of DNA wound around the eight histones. The chromatin accessibility indicating active or repressed states of genomic regions is highly correlated with the dynamics of the gene regulatory network (GRN) [26]. Notably, although only a small fraction (about 2%–3%) of the nuclear genome is kept free from nucleosomes, the accessible genome captures over 90% of regions bound by transcription factors (TFs), and enriches a large number of regulatory DNA elements such as promoters, enhancers, silencers, insulators, and genetic variants associated with diseases [27]. In recent years, multiple SCO technologies have been developed to measure chromatin accessibility by quantifying the susceptibility of chromatin to the cleavage of its constituent DNA via enzymes such as Tn5 transposase [28], [29], [30], [31], [32], [33], DNase I [34], or MNase [35]. Among these strategies, single-cell assay for transposase-accessible chromatin using sequencing (ATAC-seq) and its adaptions have been the most widely used, as they leverage hyperactive Tn5 transposons to simultaneously insert, fragment, and add Illumina sequencing adaptors to accessible chromatin regions in individual cells, achieving low cost and high accessibility for users [26].

The three-dimension genomics seeks to figure out how the chromatin of two meters in length is spatially organized into high-order structures within the micron-level nucleus, and how these architectures mediate gene expression modulations by regulatory elements [36]. At present, the main strategies for identifying single-cell genome structures are microscopy and single-cell high-throughput chromosome conformation capture (scHi-C). The microscope is able to detect a broad range of genomic interactions in a single cell, but is generally limited in terms of coverage and overall throughput [37]. In contrast, scHi-C provides the capability to interrogate genome-wide nuclear structures within a cell. In the past decade, several scHi-C methods have been developed to identify the genome-wide interactions by coupling proximity-based ligation followed by massively parallel sequencing [38], [39], [40], [41], [42], [43], [44], [45]. Additionally, a method called singe-cell SPRITE measures higher-resolution, multiway DNA contacts than that can be achieved by scHi-C [46].

The histone post-translational modification represents another epigenetic regulation, in which proteins of diverse modifications modulate the behavior of DNA molecules by physical interactions [47]. The chromatin immunoprecipitation (ChIP) is a popular method for detecting genome-wide modifications of histones and bindings of TFs. Although having been applied to identify heterogeneity of chromatin states, single-cell ChIP sequencing assays usually suffer from weak signal-to-noise ratio [48], [49], [50]. In recent years, a few non-immunoprecipitation, enzyme-tethering chromatin profiling approaches have been developed to improve the efficiency of epigenomic analysis at the single-cell level. For example, scCUT&Tag [51], CoBATCH [52], ACT-seq [53], and ChIL-seq [54] use the Tn5 transposase that is tethered to protein A binding to the antibody to simplify experimental procedures and alleviate the loss of biological signal. Additionally, the newly reported MulTI-Tag can profile multiple histone modifications simultaneously in single cells [55].

The process of DNA methylation is the covalent addition of a methyl (CH3) group to the 5′-carbon of the cytosine to form 5-methylcytosine. The DNA methylation occurs almost exclusively in the context of CpG dinucleotides of the genome and plays an essential role in gene regulation via recruiting proteins repressing transcription or inhibiting the binding of TFs [56]. Single-cell genome-wide DNA methylation sequencing methods mainly apply two strategies: reduced-representation bisulfite sequencing [57], [58] or whole-genome bisulfite sequencing [59], [60], [61]. Recently, a single-cell extended-representation bisulfite sequencing technology struck a balance between the coverage and enrichment of regulatory elements [62]. Importantly, it has been revealed that DNA modifications may also have long-term noninvasive lineage-tracking potential for their inheritance [25]. In addition, the dynamics of DNA methylation have shown great association with mammalian aging, and specific CpG loci can predict the biological ages of cells, tissues, as well as organisms [63], [64].

### Transcriptome sequencing at the center stage of SCO

With tremendous advances made in the throughput, accuracy, automation, and commercialization, scRNA-seq has been the most widely used single-cell option because of its low cost and high availability for most researchers. Two complementary strategies are frequently used [65]. Plate-based methods usually capture full-length messenger RNA (mRNA), and can detect more genes and transcripts including low abundance transcripts as well as alternative splicing events in individual cells [66], [67], [68], [69]. In contrast, droplet-based approaches [70], [71] are more likely to detect rare cell types given their properties of high throughput. For example, the 10X Genomics system can partition up to 10,000 cells per channel [72]. However, this kind of method is usually subjected to more dropout events since only a fraction of the transcriptome is captured in individual cells [73]. Additionally, most scRNA-seq approaches only provide snapshots of cellular transcriptomes rather than temporal dynamics. Several methods have come a long way to distinguish newly transcribed and pre-existing mRNAs in the same cell [74], [75], [76], [77]. The newly reported Live-seq enables the single-cell transcriptome profiling while keeping the cell alive and functional [78].

### All in one: single-cell multimodal omics

Despite great advances in profiling cell-to-cell heterogeneity at unprecedented resolution and scale, these technologies can only investigate one characteristic of cells. In fact, molecular interrogations of single cells encompass not only the genome, epigenome, and transcriptome, but also epitranscriptome [79], [80], proteome [81], as well as epiproteome [82], all of which collectively depict comprehensive characteristics of cells. In order to dissect a cell more comprehensively, single-cell multimodal omics sequencing tools [83], [84], [85], [86], [87], [88], [89], [90], [91], [92], [93], [94], [95], [96], [97], [98], [99], [100], [101], [102], [103], [104], [105], [106], [107], [108], [109], [110], [111], [112], [113], [114], [115], [116], [117], [118], [119], [120], [121], [122], [123], [124], [125], [126], [127], [128], [129], [130], [131], [132], [133], [134], [135], [136], [137], [138], [139], [140], [141], [142], [143], [144], [145], [146], [147] emerge by measuring multiple modalities at a time in an individual cell (Table 1). Importantly, combining SCO sequencing with clustered regularly interspaced short palindromic repeats (CRISPR)-based perturbations can directly associate genotypes with phenotypes. These have greatly accelerated our comprehensive understanding of the complexity of genetic variants, gene expression, intracellular regulatory networks, intercellular crosstalk, and environmental effects on animal cells.

**Table 1:** **Single-cell multimodal omics sequencing methods**

**Method**	**Genome**	**Chromatin accessibility**	**DNA methylation**	**Histone modification**	**Chromosome conformation**	**Transcriptome**	**Proteome**	**Perturbation**	**Ref.**
DR-seq	✓					✓			[83]
G&T-seq	✓					✓			[85]
SCTG	✓					✓			[84]
sci-L3-RNA/DNA	✓					✓			[116]
scONE-seq	✓					✓			[147]
SIDR-seq	✓					✓			[103]
TARGET-seq	✓					✓			[113]
DNTR-seq	✓					✓			[120]
scNanoATAC-seq	✓	✓							[146]
scGET-seq	✓	✓							[142]
scTrio-seq	✓		✓			✓			[91]
scTrio-seq2	✓		✓			✓			[99]
scCOOL-seq	✓	✓	✓						[95]
iscCOOL-seq	✓	✓	✓						[107]
PHAGE-ATAC	✓	✓					✓		[137]
sci-CAR		✓				✓			[100]
scPCOR-seq		✓				✓			[139]
scTHS-seq		✓				✓			[104]
SNARE-seq		✓				✓			[105]
SNARE-seq2		✓				✓			[127]
scCAT-seq		✓				✓			[110]
Paired-seq		✓				✓			[117]
SHARE-seq		✓				✓			[118]
ASTAR-seq		✓				✓			[119]
ISSAAC-seq		✓				✓			[144]
scNOMe-seq		✓	✓						[97]
nano-CT		✓		✓					[135]
Pi-ATAC		✓					✓		[101]
ASAP-seq		✓					✓		[125]
ICICLE-seq		✓					✓		[130]
scNOMeRe-seq		✓	✓			✓			[131]
scChaRM-seq		✓	✓			✓			[133]
snmCAT-seq		✓	✓			✓			[138]
scNMT-seq		✓	✓			✓			[102]
DOGMA-seq		✓				✓	✓		[125]
NEAT-seq		✓				✓	✓		[136]
TEA-seq		✓				✓	✓		[130]
scM&T-seq			✓			✓			[86]
sc-GEM			✓			✓			[87]
scMT-seq			✓			✓			[92]
Smart-RRBS			✓			✓			[122]
sn-m3C-seq			✓		✓				[108]
scMethyl-HiC			✓		✓				[109]
scDam&T-seq				✓		✓			[114]
EpiDamID with scDam&T-seq				✓		✓			[140]
CoTECH				✓		✓			[132]
Paired-Tag				✓		✓			[134]
scSET-seq				✓		✓			[129]
scNTT-seq				✓			✓		[141]
scCUT&Tag-pro				✓			✓		[145]
ORCA					✓	✓			[111]
PLAYR						✓	✓		[89]
PEA/STA						✓	✓		[90]
REAP-seq						✓	✓		[96]
CITE-seq						✓	✓		[98]
inCITE-seq						✓	✓		[121]
RAID						✓	✓		[106]
SCITO-seq						✓	✓		[123]
SPARC						✓	✓		[128]
Prox-seq						✓	✓		[143]
Perturb-seq						✓		✓	[88]
CRISP-seq						✓		✓	[93]
CROP-seq						✓		✓	[94]
Perturb-ATAC		✓						✓	[115]
CRISPR-sciATAC		✓						✓	[124]
Spear-ATAC		✓						✓	[126]
ECCITE-seq						✓	✓	✓	[112]

*Note*: DR-seq, gDNA–mRNA sequencing; gDNA, genomic DNA; mRNA, messenger RNA; G&T-seq, genome and transcriptome sequencing; SCTG, single-cell transcriptogenomics; sci-L3, a single-cell sequencing method that combines combinatorial (3-level) indexing and linear amplification; SIDR-seq, simultaneous isolation of genomic DNA and total RNA sequencing; DNTR-seq, direct nuclear tagmentation and RNA sequencing; ATAC-seq, assay for transposase accessible chromatin with high throughput sequencing; scNanoATAC-seq, single-cell ATAC-seq on Nanopore sequencing platform; scGET-seq, single-cell genome and epigenome by transposases sequencing; scTrio-seq, single-cell triple omics sequencing; scCOOL-seq, single-cell chromatin overall omic-scale landscape sequencing; iscCOOL-seq, improved scCOOL-seq; sci-CAR, single-cell combinatorial indexing-based coassay for chromatin accessibility and mRNA; scPCOR-seq, single-cell profiling of chromatin occupancy and RNAs sequencing; scTHS-seq, single-cell transposome hypersensitive site sequencing; SNARE-seq, single-nucleus chromatin accessibility and mRNA expression sequencing; scCAT-seq, single-cell chromatin accessibility and transcriptome sequencing; Paired-seq, parallel analysis of individual cells for RNA expression and DNA accessibility by sequencing; SHARE-seq, simultaneous high-throughput ATAC and RNA expression with sequencing; ASTAR-seq, assay for single-cell transcriptome and accessibility regions with sequencing; ISSAAC-seq, *in situ* sequencing hetero RNA–DNA-hybrid after assay for transposase-accessible chromatin-sequencing; scNOMe-seq, single-cell nucleosome occupancy and methylome-sequencing; nano-CT, nano-CUT&Tag; CUT&Tag, cleavage under targets and tagmentation; Pi-ATAC, protein-indexed assay of transposase accessible chromatin with sequencing; ASAP-seq, ATAC with select antigen profiling by sequencing; ICICLE-seq, integrated cellular indexing of chromatin landscape and epitopes; scNOMeRe-seq, single-cell nucleosome occupancy, methylome, and RNA expression sequencing; scChaRM-seq, single-cell chromatin accessibility, RNA barcoding, and DNA methylation sequencing; snmCAT-seq, single-nucleus methylcytosine, chromatin accessibility, and transcriptome sequencing; scNMT-seq, single-cell nucleosome, methylation and transcription sequencing; NEAT-seq, sequencing of nuclear protein epitope abundance, chromatin accessibility and the transcriptome in single cells; TEA-seq, assay for transcription, epitopes, and accessibility with sequencing; scM&T-seq, single-cell genome-wide methylome and transcriptome sequencing; sc-GEM, single-cell analysis of genotype, expression and methylation; scMT-seq, single-cell methylome and transcriptome sequencing; RRBS, reduced representation bisulfite sequencing; sn-m3C-seq, single-nucleus methyl-3C sequencing; scMethyl-HiC, single-cell methyl-HiC; HiC, high-throughput chromosome conformation capture; scDam&T-seq, combining single-cell DNA adenine methyltransferase identification (DamID) with messenger RNA sequencing of the same cell; EpiDamID, an extension of DamID to epigenetic chromatin marks; CoTECH, combined assay of transcriptome and enriched chromatin binding; Paired-Tag, parallel analysis of individual cells for RNA expression and DNA from targeted tagmentation by sequencing; SET-seq, same cell epigenome and transcriptome sequencing; scSET-seq, single-cell SET-seq; scNTT-seq, single-cell nanobody-tethered transposition followed by sequencing; scCUT&Tag, sing-cell cleavage under targets and tagmentation; ORCA, optical reconstruction of chromatin architecture; PLAYR, proximity ligation assay for RNA; PEA/STA, proximity extension assays/specific (RNA) target amplification; REAP-seq, RNA expression and protein sequencing assay; CITE-seq, cellular indexing of transcriptomes and epitopes by sequencing; inCITE-seq, intranuclear CITE-seq; RAID, RNA and immunodetection; SCITO-seq, single-cell combinatorial indexed cytometry sequencing; SPARC, single-cell protein and RNA co-profiling; Prox-seq, proximity sequencing; CRISPR, clustered regularly interspaced short palindromic repeats; CRISP-seq, an integrated method that combines the resolution of massively parallel single-cell RNA sequencing with the genome editing scale of pooled CRISPR screens; CROP-seq, CRISPR droplet sequencing; CRISPR-sciATAC, CRISPR-based, single-cell combinatorial indexing ATAC; Spear-ATAC, single-cell perturbations with an accessibility read-out using scATAC-seq; ECCITE-seq, expanded CRISPR-compatible cellular indexing of transcriptomes and epitopes by sequencing.

## Basic pipeline of scRNA-seq data analysis

Recently, the rapid generation of SCO sequencing data and construction of cell atlases have been both the beneficiary and the driving force of computational advances, formulating a positive feedback loop. Featuring wide applicability and availability, scRNA-seq has been the center of attention for computational biologists. The total number of scRNA-seq analytic tools has reached approximately 1400 [148]. A recent review comprehensively introduced the single-cell analysis across modalities, providing suggestions for the best computational workflows for other omics data analysis including chromatin accessibility, surface protein expression, immune receptor repertoires, and spatial localization patterns [149]. In view of this, here we take scRNA-seq data analysis as an example to illustrate the computational aspect of SCO sequencing.

Although the growing number of tools substantially facilitate the inception of new scientific insights, it makes the standardization of the workflow challenging and complex. Generally, benchmarks provide suggestions for best practices to choose from. Of note, there is not always a “golden rule”. In practice, the optimal choice of computational methods could be different in the context of specific biological backgrounds and analytical goals. Researchers should make special effort to try multiple methods, perform careful parameter tuning, and sometimes tailor the algorithm according to their needs.

**Figure 2:**
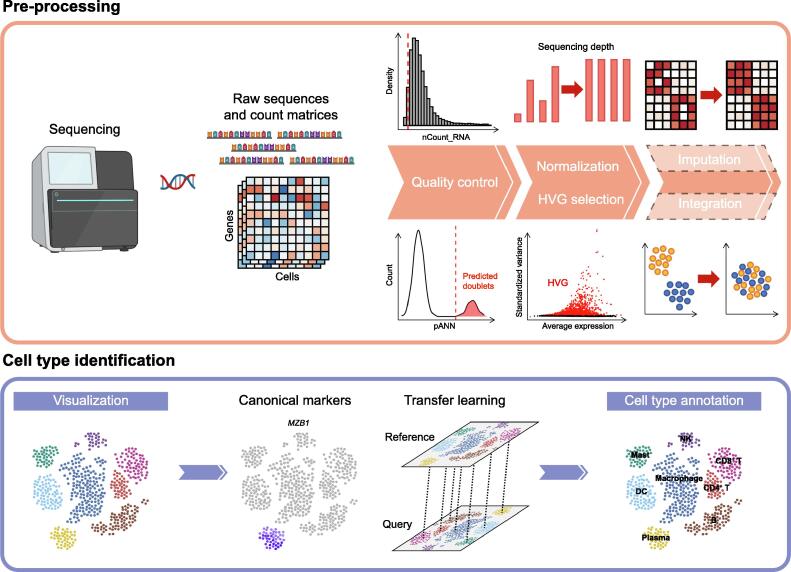
**Basic analysis in the scRNA-seq dataanalysis workflow** scRNA-seq data produced by sequencers undergo pre-processing steps including quality control, normalization, HVG selection, optional imputation, and integration. Dimensionality-reduced data are then visualized and clustered, ready to be assigned cell types with manual or automatic approaches. scRNA-seq, single-cell RNA sequencing; pANN, proportion of artificial nearest neighbors; HVG, highly variable gene; NK, natural killer; DC, dendritic cell.

Here we present an overview of the basic pipeline of scRNA-seq data analysis, from pre-processing to cell type identification (Figure 2). In each section, we summarize the conceptual reasoning, computational challenges, common practices, and recent advances.

### Quality control

After count matrices are obtained from initial raw data pre-processing, which can be readily handled by pipelines such as Cell Ranger [72] and kallisto | bustools [150], quality control (QC) is a necessary step to filter out low-quality cells, empty droplets, doublets, or multiplets. Commonly used QC metrics include the detected gene number, the total count, and the percentage of counts attributed to mitochondrial RNA in each cell. Low-quality cells and empty droplets can be identified by too few genes and total counts. High mitochondrial RNA percentage, reflecting plasma mRNA leakage due to compromised cell membrane integrity, is another sign of low-quality or dying cells. Aberrantly high gene numbers and total counts indicate doublets. Doublets can be further verified with computational doublet detection methods [151], [152], [153], most of which can be summarized as setting a threshold for the similarity of each droplet to artificial doublets. DoubletFinder [151] is considered to have the best doublet detection accuracy according to a benchmark [154]. The aforementioned QC metrics should be jointly considered and thresholds should be chosen carefully to avoid filtering out biologically informative cells. For example, comparably high mitochondrial RNA may also correspond to cells active in respiration [155].

### Normalization

The goal of normalization is to account for unbalanced sequencing depths and to stabilize the variance. Generally, normalization includes two steps, scaling with a size factor to make absolute counts comparable, and transformation to mitigate the skewness in the distribution of gene expression. The most commonly used method is LogNormalize, as implemented in Seurat [156] and SCANPY [157]. It employs a uniform size factor for all cells and performs log transformation. Evidently, it relies on the questionable assumption that all cells have the same amount of mRNA and all genes should apply the same size factor. To address this, SCnorm has been developed, which groups genes with their count–depth relationships calculated by quantile regression, and assigns each gene group a scale factor [158]. Alternatively, the increasingly popular sctransform utilizes a generalized linear model to explain the sequencing depth with counts, and performs transformation using the Pearson residuals in a gene-specific manner, although it is only applicable to unique molecular identifier (UMI)-based scRNA-seq data [159].

### Feature selection

Selecting a subset of the most informative and representative genes is a crucial prerequisite for dimensionality reduction. To reflect the variability in the expression profile across cells, the task equals to identifying highly variable genes (HVGs). Feature selection methods differ in the choice of variability measurement, including variance [156], [157], [160], [161], dropout rate [162], [163], and Gini index [164]. In the commonly used HVG selection method implemented in Seurat [156], genes are first binned according to mean expression, and HVGs are subsequently selected by normalized dispersion within each bin, thus preventing the under-representation of lowly expressed genes in HVGs.

### Imputation

It has been reported that scRNA-seq count data often exhibit a high proportion of zero values, sometimes exceeding 90% [165]. Such vast zeros raise concerns about technical noise hindering downstream analysis, for example, obscuring the gene expression correlation [166]. Methods have been invented to impute and denoise the highly sparse count data, most of which not only correct zero values, but also smooth over non-zero values, reasoning that technical noises affect all genes [167]. Some methods like MAGIC [166] treat all zeros as missing data. Conversely, methods like scImpute [168], SAVER [169], and ALRA [170] try to discriminate technical zeros from biological zeros to preserve biologically relevant information. Biological zeros originate from two sources. First, some genes are not expressed in certain cells, and second, the transcription process is not constant but intermittent, in waves of bursts, thus generating transient zero expression [171]. Of note, the imputation step is not always necessary in the scRNA-seq pre-processing workflow. It has been reported that UMI-based scRNA-seq count data are not zero-inflated, thus nullifying the necessity for imputation [172], [173]. In brief, UMI-based counts are less susceptible to amplification bias, greatly lowering the threshold for lowly expressed genes such that they become non-zero, and can be sufficiently modeled with negative binomial distribution without zero-inflation. Of note, over-correction may introduce false signals. An alternative strategy to address the sparsity of scRNA-seq count data is to pool individual cells with similar phenotypes into small groups called metacells, which still offer higher intragroup homogeneity and inter-group granularity than unsupervised clusters, as implemented in MetaCell [174] and SEACells [175]. Nevertheless, one major concern of metacells is the possibility of underrepresentation for rare cell types or cell states, and the metacell-based strategies have not been routinely applied in scRNA-seq data analysis.

### Integration

Batch effects, which represent technical variation arising from experimental procedures and sometimes include biological factors such as tissues or species [176], can potentially overshadow relevant biological signals. Batch effects almost universally exist in scRNA-seq datasets, and their complexity escalates exponentially when performing atlas-level integration, posing a grand challenge. The goal of integration is to minimize the undesired batch effects, while preserving informative biological variability. Measuring tradeoffs between batch correction and biological variance conservation, scIB [176] compared the overall performance of 16 integration methods in different scenarios, and concluded that a linear embedding model Harmony [177] performs well on datasets with simple and distinct batches, whereas a mutual nearest neighbor matching-based method Scanorama [178] and deep learning-based methods like scVI [161], scANVI [179], and scGen [180], perform best in complex tasks. Deep learning-based methods, most based on autoencoder networks, dominate atlas-level integration, which can be partly attributed to their flexible tunability to reflect complex structures of batch effects, although sometimes at the cost of decreased interpretability and increased time/space complexity.

### Cell type identification

For visualization convenience, feature-selected data undergo non-linear dimensionality reduction, either t-distributed stochastic neighbor embedding (t-SNE) [181] or uniform manifold approximation and projection (UMAP) [182], with the latter excelling at time consumption, reproducibility, and global structure preservation [183]. Cells are clustered subsequently, during which process the purity of putative clusters can be evaluated by an entropy-based metric provided by ROGUE [184] for the tuning of clustering resolution. For the subsequent cell type assignment stage, the annotation strategies can be divided into manual and automatic approaches. The manual approach relies on the observation of expression hotspots of canonical markers, which can be obtained from databases such as CellMarker [185] and PanglaoDB [186], or from the literature. Sometimes rounds of sub-clustering are needed to discover rare subsets. Although labor-intensive and subjective, the manual curation process is flexible and tunable in terms of annotation resolution. Conversely, the automatic approaches are time-saving and heavily rely on reference datasets, including marker-based CellAssign [187] and scSorter [188], correlation-based SingleR [189], and supervised classification-based SciBet [190]. Recently, the construction of large atlases has inspired the invention of new annotation methods, including CellTypist [191], scArches [192], TOSICA [193], and scBERT [194]. In essence, scArches, TOSICA, and scBERT are deep learning-based, resolving the annotation problem with query-to-reference integration by transfer learning, allowing the easy reuse of annotated consortia without sharing raw data.

## Advanced analysis of scRNA-seq data

**Figure 3:**
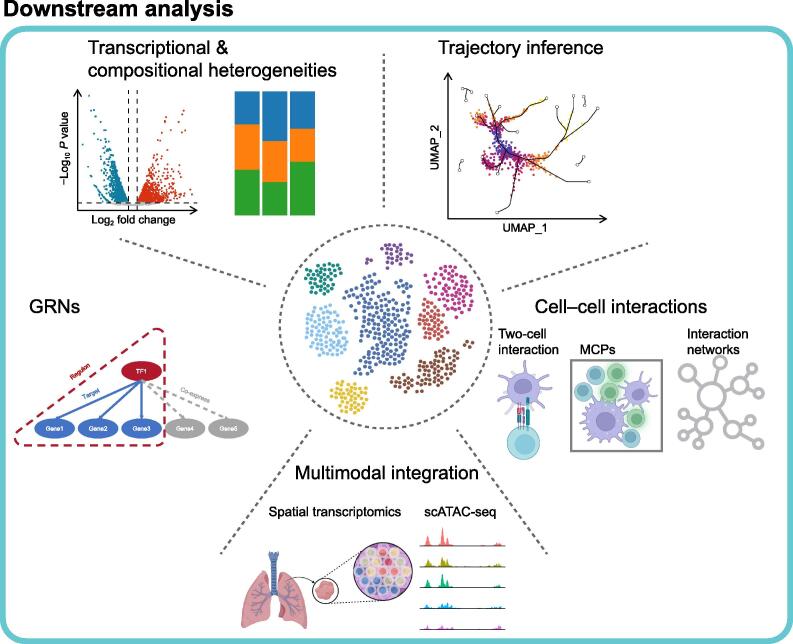
**Advanced downstream analysis in the scRNA-seq dataanalysis workflow** Downstream analysis of scRNA-seq data includes transcriptional and compositional comparative analysis, trajectory inference, GRN reconstruction, cell–cell interaction exploration, and multimodal integration. UMAP, uniform manifold approximation and projection; GRN, gene regulatory network; TF, transcription factor, MCP, multicellular program.

In contrast to the relatively fixed workflow of basic analysis, advanced downstream analysis of scRNA-seq features considerable flexibility and mutual complementarity, posing even greater challenges for evaluation and standardization. Here we list five popular topics (Figure 3) to illustrate, from a computational perspective, how biological questions can be answered by scRNA-seq, and how such methods may evolve.

### Transcriptional & compositional heterogeneities

The most basic downstream analysis is a comparative analysis, between subpopulations or between the same subpopulation in different conditions. Transcriptional and compositional heterogeneities are two complementary facets of the comparative analysis, which are occasionally interchangeable depending on the resolution of annotations [195].

The transcriptional heterogeneity can be explored first with differential expression (DE) analysis. DE genes could be fed into downstream pathway enrichment analysis to gain functional interpretation. A primary principle of DE analysis is to account for the intrinsic biological variability to minimize false positives. Methods designed for bulk DE analysis sometimes perform ironically better than those specifically designed for scRNA-seq [196], [197]. Nevertheless, the most popular method is still the simple Wilcoxon rank-sum test, used in 86% of studies of single-cell transcriptomics [197].

One challenge of compositional analysis is that the proportions of different cell types are not independent. Significant expansion of one cell type could result in significant proportion shrinkage of all other cell types. Thus, commonly used univariate tests such as the *t*-test and the Wilcoxon rank-sum test potentially risk false positives. Recent methods scCODA [198] and Cacoa [195] resolve this problem, via joint modeling of all cell types and via isometric log-ratio transformation, respectively, to minimize the dependency among cell type proportions.

### Trajectory inference

The lineage trajectory of cells could be inferred from scRNA-seq data, tracing cell transition from one developmental stage or cell state to another, under the assumption that cells along such a trajectory are sufficiently captured and are continuous in expression profiles. Meanwhile, pseudo-temporal ordering of cells along the trajectory empowers the exploration of transition-dependent expression shift. The choice of trajectory inference methods is mainly driven by the expected trajectory topology, including the choices between connected or disconnected graphs, cycles or trees, and linearity or bifurcation [199]. Commonly used trajectory inference methods include Monocle [200], Slingshot [201], and PAGA [202]. Additional cell transition information could be obtained from the splicing maturation process of mRNA, inspiring the invention of RNA velocity [203], which describes the time derivative of gene expression state, by calculating the ratio between spliced mRNA and unspliced mRNA. The field of trajectory inference has been extended by recent advances. scVelo [203], CellRank [204], and UniTVelo [205] all combine the RNA velocity information with expression profiles to perform trajectory inference or predict cell fates, thus allowing the generation of directed trajectories and nullifying the need for choosing a root node. In addition, researchers have also attempted to align and integrate trajectories from different datasets, leading to the invention of cellAlign [206] and CAPITAL [207].

### GRNs

A key notion of gene regulation is that genes do not function in isolation. Instead, genes and TFs are organized in regulons, which constitute complex GRNs, governing and coordinating transcriptional activity of the whole genome. One popular tool for delineation of GRNs is SCENIC [208], which identifies and scores the activity of regulons, followed by the prediction of cell states based on the shared activity of regulatory subnetworks. In terms of performance, methods that do not require pseudo-time-ordered cells as input have been reported to be generally more accurate [209], such as mutual information-based PIDC [210] and random forest-based GENIE3 [211], with the latter applied in SCENIC for the identification of genes co-expressing with TFs. Nevertheless, two benchmarks independently concluded that the performance of all tested methods is less than ideal in accuracy and reproducibility [209], [212], calling for future development of better alternatives. Possible improvements have been proposed recently, such as using higher-order moments to distinguish between correlation and regulation [213]. Alternatively, methods leveraging two modalities, transcriptome and epigenome, have been invented to perform integrated regulatory analysis with improved accuracy and reproducibility, including SCENIC+ [214], GRaNIE [215], Pando [216], FigR [217], and MIRA [218]. By directly incorporating TF binding information, these methods allow the inference of enhancer-driven GRNs and the delineation of regulatory circuitry underlying the developmental trajectories of cells.

### Cell–cell interactions

Cell–cell interactions are crucial in various cell activities, including differentiation, migration, and apoptosis. Cellular interaction networks could be delineated from scRNA-seq data at different granularity, starting from interactions between two cells as building blocks. Current methods for evaluating cell–cell interactions could be divided into three categories: ligand–receptor expression-based, downstream signaling-derived, and spatial reconstruction-oriented.

Ligand–receptor expression-based methods include the commonly used CellPhoneDB [219] and recently published CellChat [220]. The former boasts the most comprehensive ligand–receptor database, and the latter employs degree metrics of graph theory to assign signaling roles, thus simplifying complex signaling patterns. In a benchmark, CellChat exhibited the highest consistency with paired spatial information among 16 cell–cell interaction tools [221].

Downstream signaling-derived methods can be represented by NicheNet [222]. NicheNet incorporates intracellular signaling, connecting the binding of a ligand–receptor pair with downstream targets. Complementary to CellPhoneDB, NicheNet empowers reverse thinking, in which pertinent DE genes are first identified and then searched for responsible upstream interactions. CytoSig [223], which measures the cytokine activity, considers downstream signaling for a different reason: the expression of cytokines and their receptors is insufficient to delineate cytokine activity due to its redundant and pleiotropic nature.

Spatial reconstruction-oriented methods like CSOmap [224] are based on the assumption that cells are assembled into spatial structures, where the cell proximity is determined by the ligand–receptor interaction strength. Therefore, CSOmap can reconstruct cell spatial organizations *de novo* from scRNA-seq data. Similarly, NovoSpaRc [225] can also perform spatial reconstruction, although by expression profile similarity instead of interaction strength between cells.

In fact, such interactions occur beyond just between two cells. Under the reasoning that cells in the same spatial niche are exposed to shared cues, eliciting coordinated expression shift, a research group defined multicellular programs (MCPs) as combinations of different cell types and their coordinated expression programs in the tissue, and developed the first method to systematically uncover MCPs, DIALOGUE [226]. We envision that the structural complexity of interaction units will be increasingly represented, leading to the ultimate full depiction of cell–cell interaction networks.

### Multimodal integration

The advantage of integration and joint analysis of multimodalities is to complement transcriptomic data with extra information to better delineate regulatory networks and cell–cell interactions.

Spatially resolved transcriptomics assays can provide spatial information lost in the dissociation step of scRNA-seq. The integration of spatial and single-cell transcriptomics includes two aspects, to predict the spatial distribution of undetected RNA transcripts and to deconvolute the cell type composition of each detected spot. As assessed in a benchmark [227], methods based on probabilistic models combined with negative binomial or Poisson distributions, such as gimVI [228], RCTD [229], and Cell2location [230], generally excel at both tasks, and the sparsity of the spatial data is a major determinant of the performance of the aforementioned methods, encouraging increasing depth of sequencing or implementing imputation methods to combat this issue.

ATAC-seq assays can confer the heterogeneity of chromatin accessibility. Although recent sequencing methods like single-nucleus chromatin accessibility and mRNA expression sequencing (SNARE-seq) [105] and simultaneous high-throughput ATAC and RNA expression with sequencing (SHARE-seq) [118] enable simultaneous profiling of transcriptome and epigenome from the same cell, different omics data are often unpaired. Methods to integrate unpaired RNA-seq and ATAC-seq data typically aim to align gene expression profiles and chromatin accessibility-derived gene activity scores in a shared space. Canonical correlation analysis (CCA)-based methods include Signac [231], LIGER [232], and bindSC [233]. Among them, bindSC takes a step further beyond traditional CCA by iteratively aligning the matrices of two modalities, thus generating refined co-embeddings. Alternatively, a shared feature representation can be obtained via the reconstruction of a common weighted nearest neighbor (WNN) graph and the subsequent supervised principal component analysis, as implemented in Seurat [156]. A tough challenge is how to resolve distinct feature spaces of different modalities with minimum information loss. The recently proposed GLUE [234] overcomes this challenge by explicitly modeling regulatory interactions across omics layers and achieves accurate and scalable integration between transcriptome and epigenome data. In contrast to the integration of unpaired multimodal data, MOFA+ [235] and MultiVI [236] are designed for the integration within the same sample space, requiring the measurements of individual modalities performed on the exact same population of cells. Typically, multimodal integration methods are applicable to diverse multimodal integration scenarios, bridging the transcriptome, surface proteome, and epigenome to comprehensively characterize distinct cell states and dynamics.

## Mapping cellular heterogeneity using SCO sequencing

### Dissecting the cellular diversity of the human body

Although there are numerous advanced SCO sequencing technologies, the most widely used one is scRNA-seq for its low cost and robust performance. In recent years, a large number of studies have delineated transcriptome characteristics of diverse cell types and cell states in both homeostatic and diseased conditions. Herein, we review a few representative insights. Deep profiling of ∼ 2400 cells from human blood using Smart-seq2 protocol identified a novel dendritic cell (DC) subset with the ability to potently activate T cells, representing less than 3% of the blood DC populations [237]. These fundamental findings have modified the taxonomy of DCs and monocytes and will facilitate their developmental and functional analysis in health and disease. For neutrophils, despite their short lifetimes and intrinsic poor viability, systematic single-cell analysis of such cells has established the reference model across multiple tissues and highlighted a discrete and definable neutrophil subpopulation expressing interferon stimulating genes [238], [239]. Another study reported a fascinating new neutrophil subset that has the ability to improve central nervous system neuron survival and axon regeneration [240]. Additionally, single-cell profiling of hematopoietic cells revealed that lineage commitment is a continuous process, challenging the classical hematopoietic model in which hematopoietic system had been acknowledged as a collection of discrete hierarchically organized progenitor populations [241].

**Figure 4:**
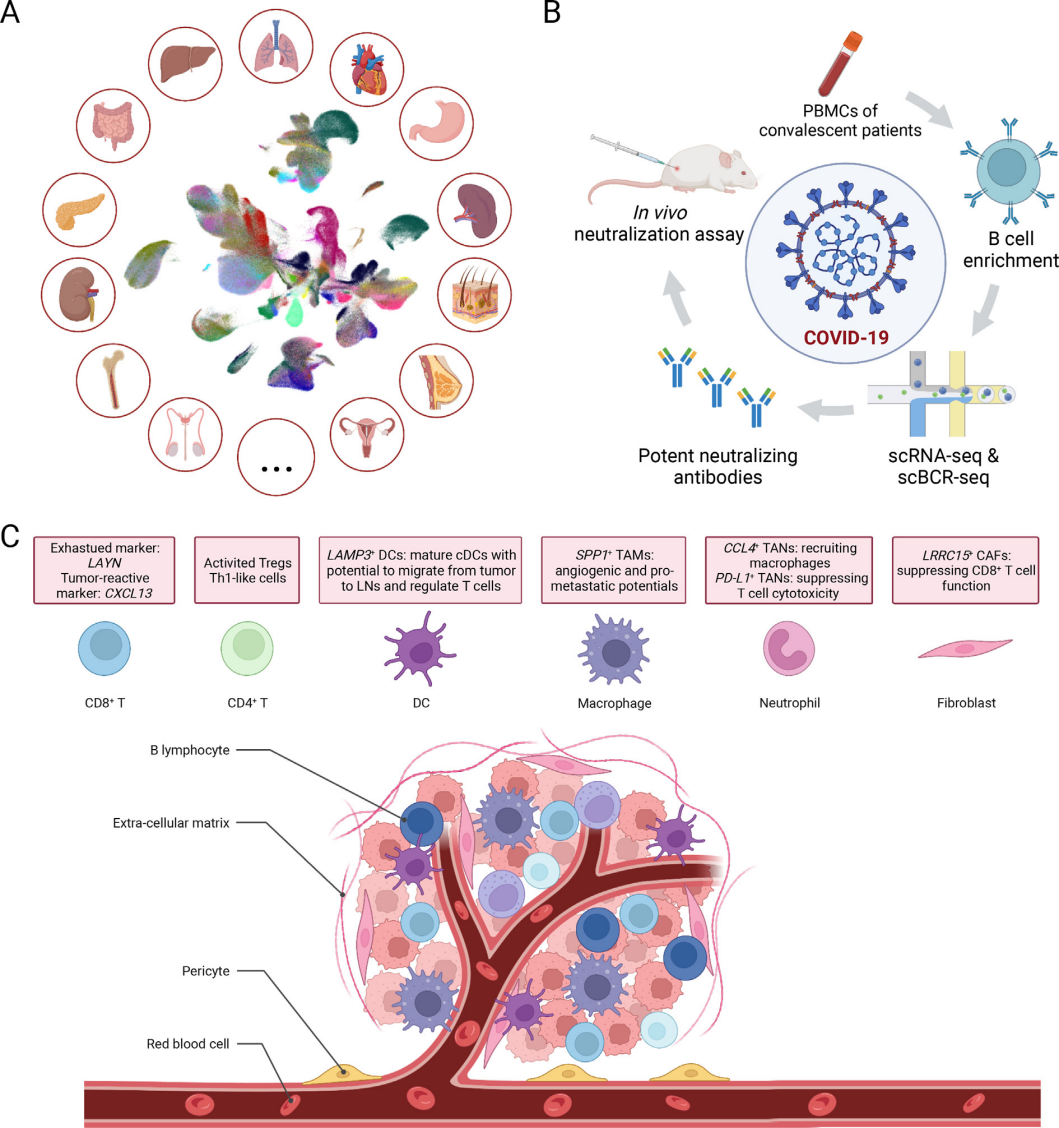
**Representative applications of SCO sequencing** **A.** A single-cell cross-tissue molecular map of the human. **B.** SCO sequencing identifies potent neutralizing antibodies in COVID-19. **C.** Dissection of TMEs using SCO sequencing. SCO, single-cell omics; COVID-19, coronavirus disease 2019; PBMC, peripheral blood mononuclear cell; scBCR-seq, single-cell B cell receptor sequencing; TME, tumor microenvironment; Treg, regulatory T cell; cDC, conventional DC; LN, lymph node; TAM, tumor-associated macrophage; TAN, tumor-associated neutrophil; CAF, cancer-associated fibroblast.

Furthermore, the ever-increasing throughput has enabled organ-level even organism-level atlases (Figure 4A). Sequencing individual nuclei and cells from six anatomical adult heart regions revealed the complexity of the cellular heterogeneity of cardiomyocytes, pericytes, as well as fibroblasts, and highlighted cardiac resident macrophages with protective and inflammatory transcriptional signatures [242]. These results deepen our understanding of the molecular mechanisms underlying cardiovascular diseases and therapeutic strategies. Profiling the spatial and temporal architecture of the developing and mature human kidney has demonstrated that the localization of antibacterial neutrophils and macrophages are well orchestrated by the epithelial-immune crosstalk to the kidney regions which are the most susceptible to infection [243]. Moreover, four back-to-back studies reported pan-tissue single-cell transcriptome atlases characterizing about 500 cell types covering more than a million cells across over 30 human tissues [191], [244], [245], [246]. Cross-tissue comparison of cell types has provided critical insights into the cell heterogeneity and revealed shared and tissue-specific transcriptional features about organ development and functions [3], [247]. Taken together, SCO technologies, especially transcriptome sequencing, have greatly facilitated the identification of rare cell types, the investigation of cellular functions, and the understanding of cell fate determinations.

### Deciphering the complexity of human disease at the cellular level

Significantly, disease-oriented applications of the SCO method have been conducted to disentangle multiple human tissues in disorder. For instance, population-scale scRNA-seq analysis of skin and blood samples from healthy controls and patients with scleroderma, a severe autoimmune disease, revealed a previously undefined scleroderma-associated fibroblast, whose perturbations are primarily associated with disease severity and clinical features [248]. A study of pediatric colitis and inflammatory bowel disease via single-cell and risk gene analysis elucidated the common pathogenesis marked by defective cyclic adenosine monophosphate (cAMP) response pathway, and demonstrated that the drug dipyridamole modulating cAMP signaling can restore immune homeostasis and improve clinical symptoms [249]. In another study, single-cell analysis of Crohn’s disease patients identified a cellular module that is composed of IgG plasma cells, inflammatory mononuclear phagocytes, activated T cells, and stromal cells, and a subset of patients highly expressing the cellular module in their inflamed tissues exhibited resistance to anti-tumor necrosis factor (TNF) therapy [250].

SCO methods have also been used to dissect the human brain, the most complex organ in the human body. For instance, compared with the normal brain, a distinct transcriptional state that corresponds to the nidus emerges in malformed human brain vasculature [251]. Profiling single-nucleus cortical transcriptomes of 48 Alzheimer’s disease individuals with varying degrees of pathology, revealed that the strongest disease-associated changes occur at early states in a cell type-specific manner, whereas genes highly expressed at late stages are common across cell types [252]. Notably, a nascent field, single-cell genetics, is emerging at the intersection of SCO and human genetics [253]. Several studies combined scRNA-seq with genotype data to identify substantial expression quantitative trait loci, most of which show cell type-specific effects on gene expression and some of which are linked to diseases [254], [255], [256], [257]. Additionally, corresponding tools have been developed to identify disease-associated cell types [258] or individual cells [259]. Taken together, these efforts have transitioned the understanding of disease biology from disease-causing genes to specific cells, programs, and tissues [260], and elucidation of the genetic basis will have broad implications for the treatment of diseases.

In the past three years, coronavirus disease 2019 (COVID-19) caused by the severe acute respiratory syndrome coronavirus 2 (SARS-CoV-2) has spread rapidly as a global pandemic. Accordingly, peripheral blood and lung biopsies of patients have been subjected to SCO sequencing around the world. The community has endeavored to analyze immune responses during COVID-19 infection to gain insights into the biology of the disease via single-cell methodologies [261], [262]. Importantly, virus neutralizing antibodies present in the plasma of convalescent patients, represent a promising therapeutic intervention by providing effective prevention for virus entry into host cells. Using high-throughput scRNA-seq and single-cell B cell receptor sequencing (scBCR-seq), potent neutralizing antibodies were identified from convalescent patients’ B cells, and their efficiencies were validated by *in vitro* and *in vivo* SARS-CoV-2 neutralization assays [263] (Figure 4B).

### Reference data resources of the wide community

In addition to great insights gained in multiple research fields, the single-cell revolution has led to the initiation and pursuit of several global consortia, projects, as well as databases (Table 2). The international Human Cell Atlas (HCA) initiative has ambitiously taken place to map all the cells of the human body [264], [265]. To date, the HCA community has generated multimodal omics data for more than 36 million cells covering nearly 5000 donors across almost all of human tissues, and all the produced datasets are being processed by uniform pipelines and publicly accessible. Beyond providing molecular cell landscape of the human body, the Human BioMolecular Atlas Program (HuBMAP) aims to build a Human Reference Atlas, which charts three-dimensional organizations of whole organs and provides standard terminologies by anatomical structures, cell types, plus biomarkers (ASCT+B) tables [266], [267]. In summary, cutting-edge SCO sequencing technologies and massive multimodal omics sequencing data are transforming our understanding of human disease at the cellular and tissue levels.

**Table 2:** **Single-cell consortia and data platforms**

**Platform**	**Web link**
Adult Human Cell Atlas	http://research.gzsums.net:8888
Allen Brain Atlas	https://portal.brain-map.org
Azimuth	https://azimuth.hubmapconsortium.org
Broad Single Cell Portal	https://singlecell.broadinstitute.org/single_cell
Cambridge Cell Atlas	https://www.cambridgecellatlas.org
CancerSEA	http://biocc.hrbmu.edu.cn/CancerSEA
COVID-19 Cell Atlas	https://www.covid19cellatlas.org
Cross-tissue Immune Cell Atlas	https://www.tissueimmunecellatlas.org
Curated Cancer Cell Atlas (3CA)	https://www.weizmann.ac.il/sites/3CA
CZ CELLxGENE Discover	https://cellxgene.cziscience.com
DISCO	https://www.immunesinglecell.org
Expression Atlas	https://www.ebi.ac.uk/gxa
FASTGenomics	https://beta.fastgenomics.org
Gene Expression Omnibus	https://www.ncbi.nlm.nih.gov/geo
GenitoUrinary Development Molecular Anatomy Project	https://www.gudmap.org
Genomic Data Commons Data Portal	https://portal.gdc.cancer.gov
Genotype-Tissue Expression project	https://gtexportal.org/home
Gut Cell Atlas	https://www.gutcellatlas.org
HCA Data Coordination Platform	https://data.humancellatlas.org
Heart Cell Atlas	https://www.heartcellatlas.org
Human BioMolecular Atlas Program	https://portal.hubmapconsortium.org
Human Cell Landscape	http://bis.zju.edu.cn/HCL/
Human Development Cell Atlas	https://developmental.cellatlas.io
Human Protein Atlas	https://www.proteinatlas.org
Human Tumor Atlas Network	https://humantumoratlas.org
HUSCH	http://husch.comp-genomics.org
JingleBells	https://jinglebells.bgu.ac.il
Kidney Cell Atlas	https://www.kidneycellatlas.org
Kidney Precision Medicine Project	https://www.kpmp.org
Liver Cell Atlas	https://livercellatlas.org
Lung Cell Atlas	https://lungcellatlas.org
LungMAP	https://www.lungmap.net
National Genomics Data Center	https://ngdc.cncb.ac.cn/databases
Oral Mucosa Cell Atlas	https://oral.cellatlas.io
PanglaoDB	https://panglaodb.se
(Re)Building a Kidney	https://rebuildingakidney.com
Reproductive Cell Atlas	https://www.reproductivecellatlas.org
SCPortalen	http://single-cell.clst.riken.jp
scRNASeqDB	https://bioinfo.uth.edu/scrnaseqdb
single-cell eQTLGen Consortium	https://eqtlgen.org/sc/index.html
Tabula Sapiens	https://tabula-sapiens-portal.ds.czbiohub.org
TISH	http://tisch.comp-genomics.org
Tissue Stability Cell Atlas	https://www.tissuestabilitycellatlas.org
UCSC Cell Browser	https://cells.ucsc.edu

## SCO sequencing in cancer research

Cancer is a systemic disease in which malignant cells arising from genetic alterations acquire capabilities for escaping from the surveillance of the immune system, proliferating uncontrollably, as well as invading local or distant normal tissues [268]. The tumor microenvironment (TME) is a complex ecosystem in which immune cells interact with heterogeneous malignant cells and stromal cells to mediate tumor progression, metastasis, relapse, and drug resistance. In the last few years, a large number of studies have been conducted to resolve the complexity of the TME at single-cell resolution and brought significant insights into TMEs across many cancer types, including melanoma [269], [270], [271], [272]; glioma [273], [274], [275]; and breast [50], [276], [277], [278], [279], [280], [281], colorectal [99], [282], [283], [284], [285], [286], [287], [288], gastric [289], [290], liver [91], [291], [292], [293], kidney [294], pancreas [295], [296], and lung [297], [298], [299], [300], [301], [302], [303], [304] cancers. Here we summarize a couple of key aspects.

### Cellular diversity in TMEs at baseline

Applications of SCO sequencing technologies have revealed a few tumor-specific cell states which could be potential targets for cancer immunotherapies to enhance anti-tumor abilities of the TME (Figure 4C). Influenced by chronic antigen stimulations, T cells usually reach a dysfunctional state called exhaustion, characterized by inactive cytotoxicity and augmented expression of inhibitory receptors, including PD-1, CTLA-4, TIM-3, TIGIT, and LAG3 [305]. Importantly, integrated analysis of tumor-infiltrating T cells from hepatocellular carcinoma patients has identified *LAYN* as a suppressive marker of exhausted CD8^+^ T cells, which are preferentially enriched and clonally expanded in tumors compared with peripheral blood and adjacent normal tissues [291]. As all T cells from the same clone share identical T cell receptor (TCR) sequences, combined scRNA-seq and single-cell TCR sequencing (scTCR-seq) can associate cellular states with clonal expansion patterns and cellular lineages [306]. For example, CD8^+^ T cells exhibiting states preceding exhaustion have also been observed in treatment-naive non-small-cell lung cancer patients, and the higher ratio of “pre-exhausted” T cells to exhausted T cells indicates better prognosis of lung adenocarcinoma [302]. Notably, combined gene expression and TCR-based lineage tracing revealed that CD8^+^ effector and exhausted T cells are independently connected with tumor-infiltrating CD8^+^ effector memory cells in colorectal cancer, although both exhibiting high clonal expansion [287]. Another player in the tumor immunity, CD4^+^ T cells, play an important role in regulating effective immune responses to cancer cells. Deep single-cell transcriptome profiling based on Smart-seq2 protocol revealed two different *FOXP3*^+^ regulatory T cells (Tregs) which present distinct distribution patterns of *TNFRSF9* (*4-1BB*) indicating activation of antigen-specific Tregs, and those activated Tregs are associated with poor prognosis in lung adenocarcinoma [302]. Notably, two T helper 1 (Th1)-like populations marked by *IFNG* have been identified in colorectal cancer, yet only *CXCL13*^+^*BHLHE40*^+^ Th1-like subset is specifically enriched in microsatellite-instable patients [287].

In addition, SCO sequencing technologies have also shed important light on the tumor-infiltrating myeloid compartment and their crosstalk with lymphocytes and non-immune cells. Integrated analysis of two scRNA-seq platforms demonstrated that *LAMP3*^+^ DCs, derived from conventional DCs (cDCs), have the potential to migrate from hepatic tumors to local lymph nodes (LNs) [293]. This population was further confirmed in lung cancer (named as ‘‘mregDC’’) [304]. Single-cell interrogations of TMEs in colorectal cancer patients revealed two distinct subsets of tumor-associated macrophages (TAMs) that show dichotomous functional phenotypes. Strikingly, the pathway analysis demonstrated that *SPP1*^+^ TAMs are specifically enriched in tumor angiogenesis genes, whereas *C1QC*^+^ TAMs are associated with the complement activation and antigen presentation signaling. Importantly, computational modeling of scRNA-seq and The Cancer Genome Atlas (TCGA) data illustrated that TAMs and cDCs constitute the core components of the cell–cell interaction network in colorectal cancer patients [288]. Recently, a large-scale single-cell atlas of human liver cancer was reported, consisting of 160 samples from 124 treatment-naive patients, all of whom can be stratified into five subtypes based on their composition of immune and stromal cells. Of the five subtypes that harbor distinct TMEs, tumor-associated neutrophils enriched in the myeloid-cell-enriched subtype have been associated with poor prognosis [292].

To understand whether such significant insights gained from a single cancer type can be extended to other cancer types, several studies conducted pan-cancer analysis to comprehensively characterize the degree of similarity and discrimination of tumor-infiltrating immune cells across different tumors. For instance, a pan-cancer study of myeloid cells showed that the proportion of mast cells varies remarkably across different cancer types. Specifically, nasopharyngeal cancer patients harbor the highest proportion of mast cells that are largely absent in many other cancer types such as multiple myeloma and hepatocellular carcinoma, indicating that mast cells might exhibit diverse functions in different tumor contexts [307]. An integrated analysis of 397,810 T cells from 316 patients across 21 cancer types, computationally detected two major developmental paths from naive to exhausted T cells, which pass through the tissue-resident memory T cells and effector memory T cells, respectively [308]. Additionally, a cross-tissue study integrating single-cell transcriptomic data of fibroblasts uncovered the cancer-associated *LRRC15*^+^ myofibroblasts, playing a role in pro-tumor immunity [4], [309]. In summary, distinguishable immune cell signatures have been found across different cancer types, which may lead to the development of more personalized immunotherapies for different patients with distinct clinical parameters.

### Insights into cancer immunotherapies

Cancer immunotherapy, attempting to restore the host’s natural defenses to eradicate malignant cells, represents a promising strategy for cancer treatment. Immune checkpoint blockades (ICBs) are designed to inhibit immunoregulatory pathways such as the PD-1/PD-L1 signaling axis, and thus to promote the elimination of malignant cells [310]. Advances in SCO sequencing technologies have enabled the comprehensive investigation of the dynamic properties of tumor-infiltrating immune cells during the course of cancer immunotherapies. Taking non-small-cell lung cancer as an example, temporal single-cell tracing analysis of 36 patients after anti-PD-1 therapy has found that precursor exhausted T (Texp) cells, which show low expression of coinhibitory genes and high expression of *GZMK*, accumulate after treatment in responsive patients. In contrast, nonresponsive patients did not exhibit the increased levels of Texp cells. In addition, paired TCR sequencing analysis has demonstrated that these Texp cells can accumulate not only through replenishment from peripheral T cells, but also through their local expansion. This has been named the clonal revival phenomenon [300]. A single-cell meta-analysis of 225 samples from 102 ICB-treated patients across five cancer types has revealed that the *CXCL13* could be an effective marker of tumor-reactive CD8^+^ T cells within tumors, and that the high proportion of *CXCL13*^+^ CD8^+^ T cells is indicative of favorable responses to ICB therapy [311]. Another study found that CD4^+^ neoantigen-reactive T cells show significant *CXCL13* expression compared with bystander cells [312]. To evaluate the efficacy of a combination of chemotherapies and immunotherapies, a triple-negative breast cancer study was conducted to dissect the dynamics of immune cells in the TME and peripheral blood of patients treated with paclitaxel or paclitaxel plus atezolizumab using scRNA-seq and single-cell ATAC sequencing. These data have revealed that the expansion of tumor-reactive immune cells caused by atezolizumab can actually be hindered by paclitaxel, leading to ineffective combination therapy [276].

Despite the success of cancer immunotherapies, durable responses have only been achieved in a fraction of cancer patients. Clearly, the dual effect of many immune cells such as macrophages and neutrophils in cancer poses a challenge for cancer immunotherapies. Specifically, the influential role of neutrophils in cancer biology and their potential as therapeutic targets are now only marginally recognized. Current cancer immunotherapies primarily target a single immune cell type or a single subpopulation of cell types. In the future, SCO sequencing technologies will play a greater role in the prediction of treatment efficiency and the development of more personalized therapies.

## Perspectives

**Figure 5:**
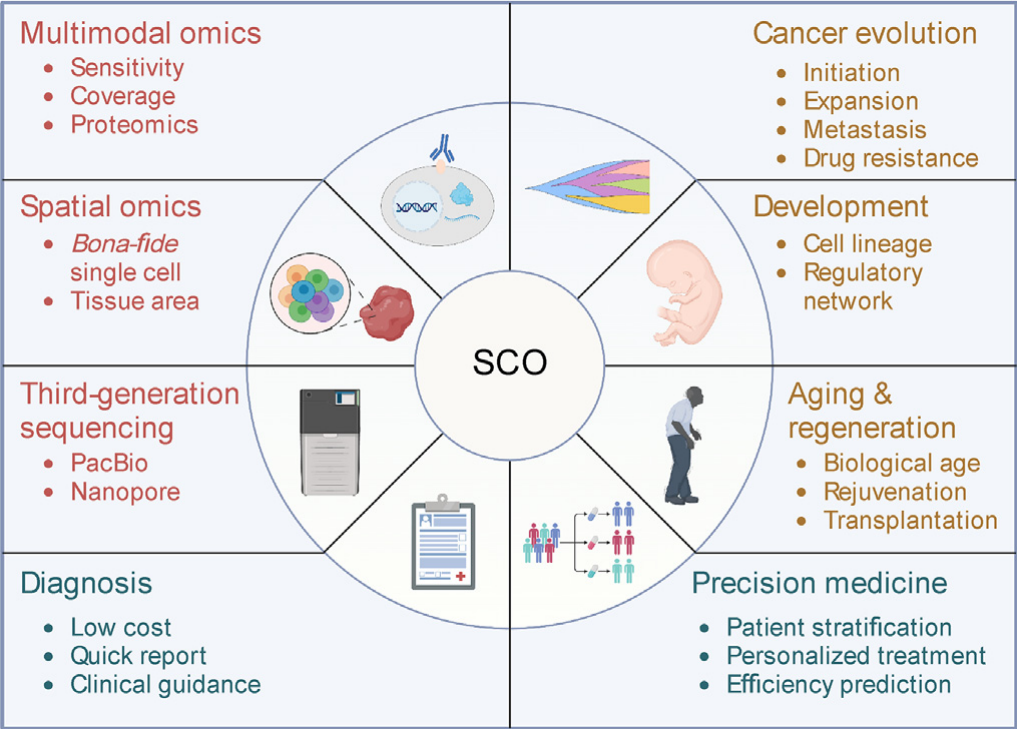
**Perspectives of SCO** Perspectives of SCO consist of technological advances, human fundamental research, and clinical prospects.

There is little doubt that the increasing use of SCO technologies in human biology and disease research will continue for the foreseeable future. This increased trend has been enabled by improvements in technologies and usability, both of which will continue to develop (Figure 5).

### Spatially resolved single-cell multimodal omics sequencing technologies

Although dozens of single-cell multimodal omics sequencing technologies have been developed (Table 1), many challenges still remain [7]. Compared to unimodal solutions, the detection sensitivity of individual modalities in single-cell multimodal sequencing methods is relatively low, which poses difficulties in distinguishing technical noise from biological signals. Additionally, these methods typically exhibit limited coverage, in the sense that not all genome-wide fragments can be captured uniformly, and thus the resulting data are too sparse to provide comprehensive characteristics of cells. Another weakness lies in the fact that few solutions have implemented single-cell high-throughput profiling of the proteome [313], which directly links the cellular output to its function. Specifically, assays for detecting genome and proteome simultaneously will enable the investigation of the extent to which genetic variants affect the physiological functions of cells and tissues. The combined analysis of transcriptome and proteome allows to delineate the dynamic relationship between RNA and protein abundance across different cell types and tissues.

Although SCO sequencing technologies have enabled the high-resolution investigations of biological systems, cell–cell spatial relationships and communication are lost during tissue dissociation. In fact, cellular functions not only depend on intracellular molecules and events, but also are tightly regulated by their well-organized niche cells [314]. Recent years have witnessed the rapid development of sequencing-based spatially resolved omics technologies [315], [316], [317], [318], [319], [320], [321], [322], [323], [324], [325], [326], [327], [328], [329], [330]. However, these spatial strategies capture partial cellular content or a mixture of multiple cells, instead of profiling cells at *bona-fide* single-cell resolution. Although the imaging-based spatially resolved methods can achieve single-cell resolution, they are still limited in terms of overall throughput and gene coverage [331]. In the future, exact single-cell spatially resolved omics sequencing may be possible by encoding cellular molecules in a self-adaption rather than grid-like fashion for individual cells. In addition, combining single-cell CRISPR-based perturbations with spatial information may enable *in situ* investigations of the complex connections between genotypes and phenotypes [325].

Finally, all current single-cell multimodal and spatially resolved omics sequencing technologies are based on next-generation sequencing platforms. To sequence long fragments directly and improve the sequencing accuracy, more high-throughput technologies based on the third-generation sequencing platforms such as Nanopore and PacBio are likely to emerge and grow.

### Decoding temporal dynamics underlying human biology and disease

In fact, most biological processes in humans are involved in temporal dynamics, such as embryo development, individual aging, and disease progression. For example, the cancer evolution consists of multiple crucial transitions, including tumor initiation from precancerous lesions to malignancy, local expansion and distant metastasis, as well as progression to the drug-resistant state [332]. In these processes, SCO sequencing technologies can play roles in unraveling the diversity of cell states, the dynamics of cell fate, and the complexity of cell–cell interactions through the sequential sampling strategies. Additionally, integrated analysis of single-cell sequencing data and a mass of archived bulk sample sequencing data with pair clinical information in the International Cancer Genome Consortium (ICGC) [333], TCGA [334], and Genotype-Tissue Expression (GTEx) [335], will reveal potential biomarkers for detecting precancerous, metastatic, and drug-resistant transitions, improve the early detection and patient stratification, and facilitate drug screening and personalized treatment.

Another threat to human health is aging, in which cells lose their physiological integrity and organs gradually display dysfunctional states. Notably, aging directly contributes to many diseases, such as neurodegeneration, cancer, and cardiovascular diseases [336]. The application of SCO sequencing technologies makes it possible to measure biological ages at the molecular and the cellular levels [337]. Calculating aging scores at the cellular level will uncover heterogeneity between cells and asynchronism between biological and chronological ages. Specifically, immune aging characterized by systemic pathogen-free inflammation in aged individuals can lead to profound effect on immune processes, and comprehensive elucidation of aging immune cells may reveal key targets to rejuvenate the immune system [338].

In addition to aging, organic diseases can also lead to functional deficiencies in organs. From a long-term perspective, transplants are the most effective treatment for organ failure. For example, human pluripotent stem cell-derived islets have been a promising strategy for the therapy of insulin-deficient diabetes [339]. The combined analysis of SCO sequencing technologies and organoids will play an important role in the exploration of stem cell maintenance and differentiation.

### Overcoming the bottleneck of translating SCO technologies to clinical applications

Significantly, SCO sequencing is beginning to profoundly impact the development of precision medicine, including more accurate patient stratification and more personalized treatment. Ultimately, translating these technological advances into clinical practice will greatly improve the accuracy of disease diagnosis and treatment. For example, functional CRISPR screens with single-cell readout will facilitate the dissection of disease mechanisms and accelerate drug discovery.

The first of many challenges that need to be addressed is the high cost associated with SCO examinations, which hinders their widespread adoption in clinical settings. A substantial reduction in cost is necessary to make single-cell-based assays affordable for most patients in a variety of diagnostic settings. One way to achieve this is through automation of sampling, library construction, sequencing, as well as medical equipment operation. Another approach is to streamline the bioinformatic analysis process via user-friendly software tools that automate data processing and analysis. In addition, it is imperative to reduce the time taken for single-cell-based assays. In general, it should take no more than two days from clinical sampling to the generation of informative medical reports for effective diagnostic or therapeutic purposes. Reducing the time and expertise required for experiments and data analysis will make single-cell assays more accessible to a wider range of healthcare. Furthermore, the vast majority of the current SCO works are of a research nature, with the primary goal of generating new hypotheses or advancing new research directions. In clinical settings, however, SCO-derived results need to provide specific guidance to medical staff members for achieving more precise diagnosis and actionable plans. Thus, it is essential to establish more definitive links between SCO-derived results and specific clinical parameters, such as treatment responses and detailed pathological classifications.

In summary, clinical desirability calls for a concerted effort by all stakeholders involved in the healthcare industry. Advances in the technology and medical research will not only improve patient outcomes but also increase the efficiency of the healthcare system. Although the road ahead will be long, SCO sequencing technologies represent a hugely promising gateway to precision medicine.

## Competing interests

Zemin Zhang is a founder of Analytical BioSciences and is a board member for InnoCare Pharma. Other authors have declared no competing interests.

## CRediT authorship contribution statement

**Qiang Shi:** Conceptualization, Investigation, Writing – original draft, Visualization, Funding acquisition. **Xueyan Chen:** Investigation, Writing – original draft, Visualization. **Zemin Zhang:** Supervision, Writing – review & editing, Funding acquisition. All authors have read and approved the final manuscript.
